# The Role of Ala198 in the Stability and Coenzyme Specificity of Bacterial Formate Dehydrogenases

**Published:** 2015

**Authors:** A. A. Alekseeva, V. V. Fedorchuk, S. A. Zarubina, E. G. Sadykhov, A. D. Matorin, S. S. Savin, V. I. Tishkov

**Affiliations:** A.N. Bach Institute of Biochemistry, Russian Academy of Sciences, Leninskiy prospect, 33/2, Moscow, 119071, Russia; Innovations and High Technologies MSU Ltd, Tsymlyanskya Str., 16–96, Moscow, 109559, Russia; Department of Chemistry, M.V. Lomonosov Moscow State University; Leninskie gory, 1/3, Moscow, 119991, Russia

**Keywords:** site-directed mutagenesis, thermal stability, coenzyme specificity, kinetic parameters

## Abstract

It has been shown by an X-ray structural analysis that the amino acid residues Ala198, which are located in the coenzyme-binding domain of NAD^+^-dependent formate dehydrogenases (EC 1.2.1.2., FDH) from bacteria* Pseudomonas *sp.101 and *Moraxella *sp. C-1 (PseFDH and MorFDH, respectively), have non-optimal values of the angles ψ and φ. These residues were replaced with Gly by site-directed mutagenesis. The mutants PseFDH A198G and MorFDH A198G were expressed in *E.coli *cells and obtained in active and soluble forms with more than 95% purity. The study of thermal inactivation kinetics showed that the mutation A198G results in a 2.5- fold increase in stability compared to one for the wild-type enzymes. Kinetic experiments indicate that A198G replacement reduces the K_M_^NAD+^ value from 60 to 35 and from 80 to 45 μM for PseFDH and MorFDH, respectively, while the K_M_^HCOO-^ value remains practically unchanged. Amino acid replacement A198G was also added to the mutant PseFDH D221S with the coenzyme specificity changed from NAD^+^ to NADP^+^. In this case, an increase in thermal stability was also observed, but the influence of the mutation on the kinetic parameters was opposite: KM increased from 190 to 280 μM and from 43 to 89 mM for NADP^+^ and formate, respectively. According to the data obtained, inference could be drawn that earlier formate dehydrogenase from bacterium *Pseudomonas *sp. 101 was specific to NADP^+^, but not to NAD^+^.

## INTRODUCTION

**Fig. 1:**
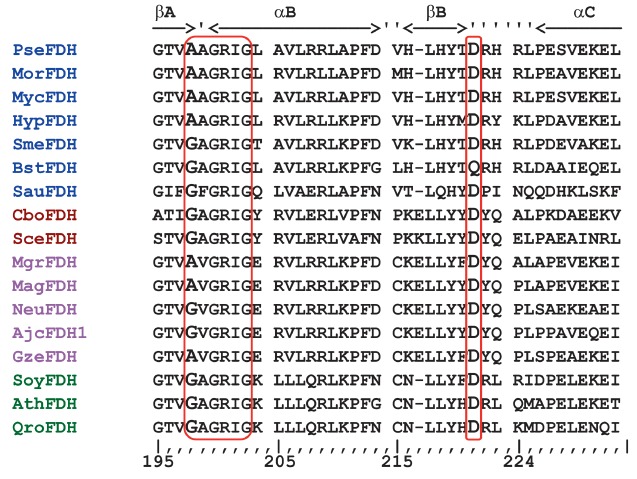
Alignment of amino acid sequences of formate dehydrogenases from different sources in the region of the coenzyme-binding domain. Bacterial FDHs are marked in blue: PseFDH –*Pseudomonas *sp.101 (UniProtKB/Swiss- Prot: P33160.3), MorFDH – *Moraxella *sp. C-1 (GenBank Accession Y13245), MycFDH –*Mycobacterium vaccae *N10 (GenBank BAB69476), HypFDH – *Hyphomicrobium strain *JC-17 [GenBank BAB55449], SmeFDH – *Sinorhizobium meliloti *16262453 (GenBank NP_435497), BstFDH – *Burkholderia stabilis *(GenBank CP000378), SauFDH – *Staphylococcus aureus *(NCBI Reference Sequence: WP_031923037.1). FDHs from yeasts are marked in brown: CboFDH –* Candida boidinii *(GenBank Accession ABE69165), SceFDH – baker’s yeast *S.cerevisiae *(EMBL Z75296). FDHs from fungi are marked in magenta: MgrFDH –*Mycosphaerella graminicola *(Septoria tritici) (UniProt Q9Y790), MagFDH –*Magnaporthe grisea *(EMBL AA415108), NeuFDH –*Neurospora crassa *[GenBank Accession XP_961202.] AjcFDH –*Ajellomyces capsulatus *[GenBank Accession XP_001539240], GzeFDH –*Gibberella zeae *(GenBank Accession XM_386303) and FDHs from plants are marked in green: SoyFDH –soybean *Glycine max *(GenBank Accession GB BT094321), AthFDH –*Arabidopsis thaliana *(EMBL AF208029), QroFDH –oak *Quercus robur *(GenBank Accession GB AJ577266)

A characteristic feature of NAD^+^-dependent dehydrogenases is the presence of the specific sequence (fingerprint) of GxGxxG in their coenzyme-binding domain [1]. In fact, the only exception to this rule is formate dehydrogenase from bacteria and fungi [EC 1.2.1.2] (FDH). In all bacterial FDHs (except for enzymes from symbiotic bacterium *Sinorhizobium meliloti *and bacteria of the genera *Bordetella *and *Staphylococcus*) the GxGxxG sequence contains the Ala, instead of Gly, residue in the first position (*Fig. 1*). A similar pattern was observed for the enzyme from fungi, whereas all known FDHs from various yeasts and plants obey the rule above and possess a classic characteristic sequence.

**Fig. 2:**
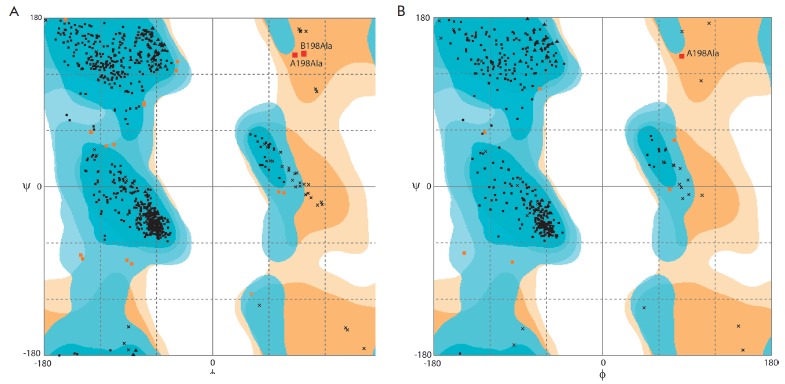
Ramachandran plot for the structures of the apo-forms of formate dehydrogenases from bacteria *Pseudomonas* sp.101 (A) (PDB2NAC) and *Moraxella *sp. C-1 (B) (PDB3FN4). Only one pair of angles ψ and φ is shown for MorFDH because the crystallographic cell of the latter enzyme contains only one enzyme subunit

**Fig. 3:**
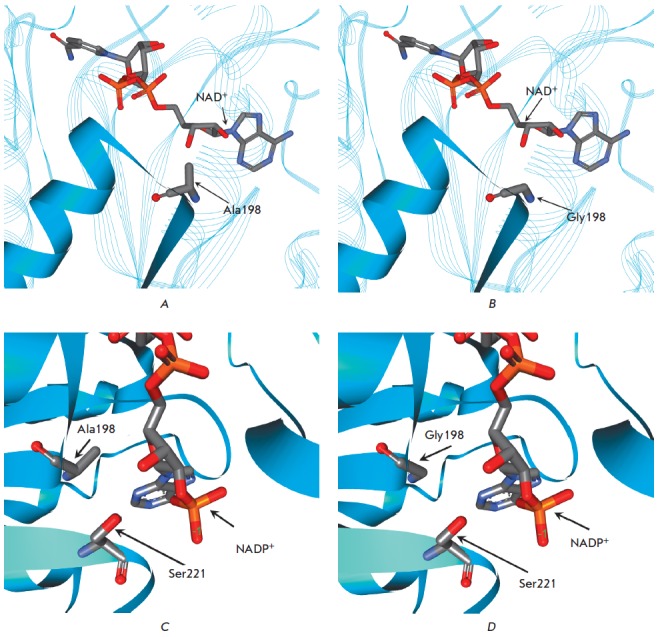
Fragments of the structures of formate dehydrogenase from bacterium *Pseudomonas *sp.101. A – wild-type enzyme (complex with NAD^+^ and azide – 2NAD), B – model structure of the mutant PseFDH A198G (complex with NAD^+^); C and D – model structures of mutant PseFDH D221S and PseFDH A198G/D221S (both – complexes with NADP^+^), respectively

In our laboratory, a systematic study of FDH from various sources, including methylotrophic bacteria* Pseudomonas *sp. 101 and *Moraxella *sp. C-1 (PseFDH and MorFDH, respectively), was performed. In both enzymes, the non-canonical Ala residue was located at position 198. In collaboration with the laboratory headed by prof. Vladimir Popov (A.N. Bach Institute of Biochemistry, Russian Academy of Sciences), and groups led by of Dr. Victor Lamzin (EMBL Outstation, Hamburg) and Dr. Konstantin Polyakov (Engelhardt Institute of Molecular Biology, Russian Academy of Sciences), three-dimensional structures of apo-forms of PseFDH and MorFDH and their various complexes were solved [2-5]. In the structures of both enzymes, the Ala198 residue has “forbidden” values for the angles ψ and φ, preventing an optimal orientation of secondary structural elements (*Fig. 2*). Analysis of the Xray structures of PseFDH MorFDH indicates that the Ala198 residue is located between the βA strand and αB helix (*Fig. 3A*).

In the present study, Ala198 was replaced with Gly by site-directed mutagenesis in order to decrease conformational tension and elucidate the role of Ala198 in the stability and catalytic properties of PseFDH and MorFDH. Additionally, the Ala198Gly mutation was introduced into PseFDH with the Asp221Ser substi- tution, which was previously obtained in our laboratory. As a result of the latter substitution, the coenzyme specificity of PseFDH changed from NAD^+^ to NADP^+^ [6, 7]; therefore, it was important to determine how the removal of conformational tension affects the stability and coenzyme specificity of NADP^+^-specific PseFDH.

## MATERIALS AND METHODS

Molecular Biology Grade reagents were used for the genetic engineering experiments. Bactotryptone, yeast extract and agar (Difco, USA), glycerol (99.9%) and calcium chloride (“ultra pure”), potassium hydrogen phosphate, sodium dihydrogen phosphate (“pure for analysis”), lysozyme (Fluka/BioChemika, Switzerland), lactose (analytical grade), ampicillin and chloramphenicol (Sigma, USA), and sodium chloride (“AR grade”, Helicon, Russia) were used in the microbiological experiments. Restriction endonucleases, DNA ligase of T4 phage, and Pfu-DNA polymerase (Thermo Scientific) were used for cloning DNA fragments and site-directed mutagenesis. Thermo Scientific reagent kits were used to isolate DNA from agarose gel and plasmids from *E. coli *cells. The oligonucleotides for the polymerase chain reaction (PCR) and sequencing were synthesized by Synthol (Russia). MilliQ (Millipore, USA) purified water was used in these experiments.

All reagents for the electrophoresis of proteins were manufactured by Bio-Rad (USA). Ammonium sulfate (chemically pure, Dia-M, Russia), urea (pure for analysis, Reahim, Russia), NAD^+^ and NADP^+^ with purity of at least 99% (AppliChem, Germany), EDTA, sodium formate and sodium dihydrogen (pure for analysis, Merck, Germany), sodium azide (Serva, Germany) were used for the purification and characterization of the enzyme.

**Site-directed mutagenesis**

Nucleotide substitutions were introduced using twostep PCR. Plasmids pPseFDH8, pPseFDH8_D221S, and pMorFDH2, with the *psefdh *and *morfdh *genes under the control of a strong promoter of T7 RNA polymerase, were used as templates. The mutations were introduced using forward (T7_For) and reverse (T7_Rev) primers at the beginning and at the end of the gene, respectively, as well as direct and reverse primers carrying the desired replacements for the *psefdh *gene. The primer sequences are shown below.

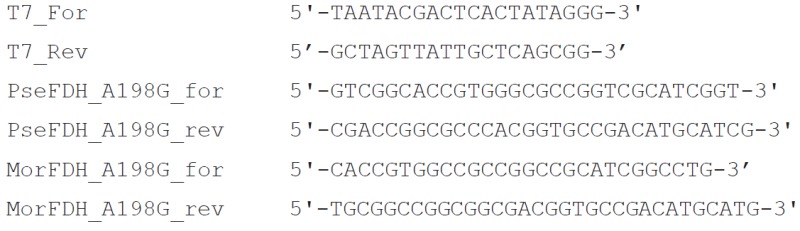



The reaction mixture for PCR contained 2.5 μl of a 10× buffer for Pfu-DNA polymerase (200 mM Tris- HCl (pH 8.8 at 25°C), 100 mM (NH_4_)_2_SO_4_, 100 mM KCl, 1 mg/ml BSA, 1% (v/v) Triton X-100, 20 mM MgSO_4_); 2.5 μl of a dNTP mixture (dATP, dGTP, dTTP, dCTP with the concentration of each component being 2.5 mM); 1 μl of the DNA template (≈10 ng/μL); 2 μl of each primer (10 nM/ml); 0.5 μl of Pfu-DNA polymerase (2.5 U/μl); and deionized water to a total volume of the mixture of 25 μl. PCR was performed in a 0.5-ml thinwalled plastic tube (SSI, USA) using a Tertsik instrument (DNA Technologies, Russia). A total of 30 μl of mineral oil was added to the tube before the PCR to prevent evaporation of the reaction mixture. The tube was heated for 5 min at 95°C, and the PCR reaction was carried out according to the following scheme: denaturation at 95°C, 30 s; primer annealing at 54–58°C; and extension at 72°C, 2 min, a total of 25–35 cycles. After the last cycle, the reaction mixture was further incubated for 10 min at 72°C. The temperature during the second step was 3–5°C below the melting temperature of the duplexes (T_m_) formed by the primers.

For the first two PCR runs, T7_For/PseFDH_ A198G_rev (fragment 1) and PseFDH_A198G_for/T7_ Rev (fragment 2) primer pairs were used in the case of PseFDH. Fragments T7_For/MorFDH_A198G_rev and MorFDH_A198G_for/T7_Rev (fragments 1 and 2, respectively) were used for MorFDH. The PCR products – fragment 1 and fragment 2 – were purified using electrophoresis in 1% agarose gel. The third uniting PCR was then performed with the T7_For and T7_Rev primers, wherein the previously obtained fragments, 1 and 2, were used as the DNA template. The product of the third PCR was purified in a similar way using 1% agarose gel and treated with two restriction endonucleases: XhoI and EcoRI. DNA fragments were purified using electrophoresis in 1% agarose gel, extracted from the gel and ligated with the plasmids pPseFDH8, pPseFDH8_D221S, and pMorFDH2, treated with the same restriction endonucleases. The ligation mixture was used to transform *E. coli *DH5α cells. The cells were then plated onto Petri dishes with an agar medium containing ampicillin (100 μg/ml) and incubated for 16 h at 37°C. Three colonies of each mutant PseFDH A198G, PseFDH A198G/D221S, and MorFDH A198G were taken from each plate and used to isolate the plasmids. The presence of only the desired mutations was proved by sequencing using the plasmid DNA at the center for the collective use “Genome” (V.A. Engelhardt Institute of Molecular Biology, Russian Academy of Sciences).

**Expression of FDH mutants in** *E. coli* **cells**

Wild-type PseFDH and MorFDH and their mutant forms were expressed in the *E. coli *cells BL21 (DE3)/ pLysS. The cells were transformed using the appropriate plasmid and plated on Petri dishes with an agar medium containing ampicillin (100 μg/ml) and chloramphenicol (25 μg/ml) in order to obtain a producer strain. A single colony was taken from the plate and cultured for 7–9 h at 30°C and 180 rpm in 5 ml of a 2YT medium (yeast extract 10 g/l, bactotryptone 16 g/l, sodium chloride 5 g/l, pH 7.0) in the presence of 100 μg/ml ampicillin and 25 μg/ml chloramphenicol until an absorbance of *A*_600_ ≈ 0.6–0.8 was reached. Then inoculate was transferred into 100 ml of 2YT medium with ampicillin (100 μg/ml) in 1 l baffled conical flasks and cultured at 30°C and 80–90 rpm until an absorbance of *A*_600_ ≈ 0.6–0.8 was reached. Enzyme expression was induced by adding lactose (300 g/l) to the medium to a final concentration of 20 g/l. After induction, the cells were cultivated for 17 h at 120 rpm. The cells were pelleted using a Beckman J-21 (USA) centrifuge (20 min, 7500 rpm, 4°C). The resulting pellet was re-suspended in a 0.1 M sodium phosphate buffer (pH 8.0) in a 1: 4 (wt.) ratio. The resulting suspension was stored at –20°C.

**Isolation and purification**

The enzymes were purified using the previously developed protocol for purifying recombinant wild-type PseFDH [8]. A cell suspension, 20% (w/v) in 0.1 M sodium phosphate buffer (pH 8.0) containing wild-type PseFDH and MorFDH and their mutants, was prepared from the resulting biomass. Final suspensions were subjected to two freeze-thaw cycles, and the cells were disrupted using a sonicator (Branson Sonifier 250, Germany) under continuous cooling. The precipitate was removed by centrifugation using a 5804R Eppendorf centrifuge (11000 rpm, 30 min), and a saturated ammonium sulfate solution was added dropwise to the supernatant to a concentration of 35% of the saturated solution. The resulting solution was incubated for several hours at 4°C. The precipitate was separated by centrifugation using a Beckman J21 centrifuge (20,000 rpm, 30 min, 4°C), and the supernatant was purified on a Phenyl Sepharose Fast Flow (Pharmacia Biotech). The protein was eluted using a linear gradient 35*–*0% of ammonium sulfate in a 0.1 M sodium phosphate buffer, pH 7.0. Active fractions were collected, and the enzyme solution was concentrated by membrane filtration using a cell with a PM-10 membrane (Amicon). The enzyme preparation was desalted by gel filtration through a Sephadex G-25 column in the same buffer. Preparation purity was monitored by analytical electrophoresis in 12% polyacrylamide gel in the presence of 0.1% sodium dodecyl sulfate on a Mini- Protean III instrument (BioRad).

**Formate dehydrogenase activity assay**

FDH activity was measured spectrophotometrically by monitoring the accumulation of NADH (NADPH) at 340 nm (ε_340_ = 6220 M^-1^ cm^-1^) on a Schimadzu UV 1601PC or UV 1800PC spectrophotometers at 30°C in a 0.1 M sodium–phosphate buffer, pH 7.0. NAD(P)^+^, and formate concentrations in the cuvette were 0.6 M and 1 mg/ml, respectively.

**Determination of Michaelis constants**

The Michaelis constants (K_M_) for NAD^+^, NADP^+^ , and formate were determined from the dependence of the reaction rate on a variable substrate concentration (0.4–6 *K*_M_) at a fixed saturation concentration of the second substrate (>15 *K*_M_). The exact concentrations of the NAD^+^ and NADP^+^ solutions were determined spectrophotometrically at 260 nm (ε260 = 17800 M^-1^ cm^-1^). The sodium formate solution was prepared by dissolving the required amount of substrate in a 0.1 M sodium phosphate buffer pH 7.0. The solution was adjusted in a volumetric flask. K_M_ values were calculated by nonlinear regression using the OriginPro 8.5 software.

**Thermal inactivation study**

The thermal stability of the enzymes was studied in a sodium phosphate buffer (0.1 M, pH 7.0) at various temperatures. A number of Eppendorf tubes (0.5 ml) with a 100 μl enzyme solution (0.2 mg/ml) were prepared for each experiment. The tubes were incubated in a water bath at different temperatures with a precision of temperature control ± 0.1oC. At fixed time intervals, a tube was transferred from the bath to ice for 5 min. The solution was then centrifuged for 3 min at 12,000 rpm using an Eppendorf 5415D centrifuge. The residual FDH activity was measured as described above. The rate constant of thermal inactivation *kin *was calculated from the slope of the linear dependence of remaining activity on time (semi-log coordinates ln(A/A_0_) – *t*) by linear regression using the Origin Pro 8.5 software.

**Computer simulation**

The structures of mutant PseFDH and MorFDH were modeled using Insight II (Accelrys), and structures of apo forms of PseFDH (PDB2NAC, resolution 2.05 A) and MorFDH (PDB3FN4, resolution 1.96 A) were used as the template structures. Further optimization of the structure was performed using molecular mechanics (module Discover_3 in Insight II, a force field CVFF, 1000 cycles), molecular dynamics (5 ps), and again molecular mechanics (1000 cycles). The PseFDH and MorFDH structures were analyzed using the Accelrys Discovery Studio 2.1 software package. The same package was used to obtain images of the protein globule.

## RESULTS AND DISCUSSION

**Selection of amino acid residues for site-directed mutagenesis**

Enzymes with the desired properties can be successfully obtained using protein engineering methods. Rational design is one of the widely used approaches. During the first stage, the three-dimensional structure of the target enzyme is analyzed and the sites of directed amino acid substitutions are identified. Multiple alignments of amino acid sequences for the site selected for the substitutions were performed in order to determine the type of introduced residues. The final choice of residues is made after analyzing the model structures of potential mutants. We used the rational design method for two formate dehydrogenases from bacteria *Pseudomonas* sp.101 and *Moraxella *sp. C-1.

The part of the active center binding the adenine portion of the coenzyme is known to have a number of structural features typical of NAD(P)+-dependent dehydrogenases. The coenzyme-binding domain consists of two subdomains binding the adenine and nicotinamide portions of the coenzyme in the majority of enzymes of this group. Each of these sub-domains is composed of alternating β-strands and α-helices. This structure is called the Rossmann fold [9]. The total amount of alternating β-strands and α-helices can be different. Various folding options were analyzed in [10]. The GxGxxG-conserved motif is located in the site connecting the first strand of the β-sheet to the α-chain of the Rossmann fold. The first glycine residue, due to its high mobility, provides optimum relative positioning of the secondary structures required for proper orientation of the second glycine residue of this motif. The second Gly residue located in the immediate vicinity of the phosphate moiety of the coenzyme is involved in its binding. It is assumed that at this position the presence of any residue with bulkier side chains would lead to strong steric complications upon binding of the coenzyme. The third residue is important for dense packing of αA and βB structural elements and their relative orientation.

The alignment of the amino acid sequences of formate dehydrogenase from different organisms in the site of the coenzyme-binding domain (fragment β-α-β) is shown in Fig. 1. This alignment demonstrates that a significant part of formate dehydrogenases from bacteria and fungi contains Ala residue at the first position instead of Gly. This Ala residue resides at position 198 in FDH from bacteria *Pseudomonas *sp. 101 and *Moraxella* sp. C-1. According to Ramachandran plots, PseFDH and MorFDH apo-forms (2NAC and 3FN4, respectively) possess non-optimal values of the angles ψ and φ of the Ala198 residue (*Fig. 2 A, B*).

As can be seen from Fig. 3A, the methyl group of the Ala residue is oriented toward the 3’-OH of ribose of adenosine in the ternary complex of PseFDH with NAD+ and the azide ion (holo form, 2NAD, resolution 1.8 A, the structure is considered to be an analogue of the transition state). The results of a computer simulation demonstrated that Ala198Gly substitution in PseFDH removes conformational tension (*Fig. 3B*). The situation is similar for MorFDH (holo form 2GSD structure, not shown in *Fig. 3*). Based on computer modeling, we decided to obtain mutant PseFDH and MorFDH, where the Ala198 residue is replaced by Gly.

A Ala198Gly substitution was also introduced in a previously obtained PseFDH mutant with the coenzyme specificity changed from NAD+ to NADP+. This effect was achieved by a Asp221Ser substitution (*Fig. 3C*). The results of a computer simulation showed (*Fig. 3C*) that steric tension in the structure of the mutant enzyme in complex with NADP+ is not as strong as that of the wild-type, due to the presence of the Ala198 residue. Nevertheless, an additional pocket, which would more effectively bind the 3’-phosphate group of the coenzyme, should appear as a result of Ala198Gly substitution (*Fig. 3D*).

**Preparation of mutant enzymes**

Three plasmids were isolated after mutagenesis using PCR for each of the three mutants: PseFDH Ala198Gly, PseFDH Ala198Gly/Asp221Ser, and MorFDH Ala198Gly. The sequencing results showed that all plasmids contained only the desired mutations in the* psefdh *and *morfdh *genes, while other nucleotide substitutions were absent. Plasmids with mutations were used to transform *E. coli *BL21 (DE3)/pLysS cells. Both mutant PseFDH and mutant MorFDH were expressed in soluble and active forms. They were isolated according to the method described in the Materials and Methods section. Their purity was at least 95% according to the results of analytical electrophoresis in a polyacrylamide gel in the presence of sodium dodecyl sulfate.

**Study of the thermal stability of mutant formate dehydrogenases**

**Fig. 4:**
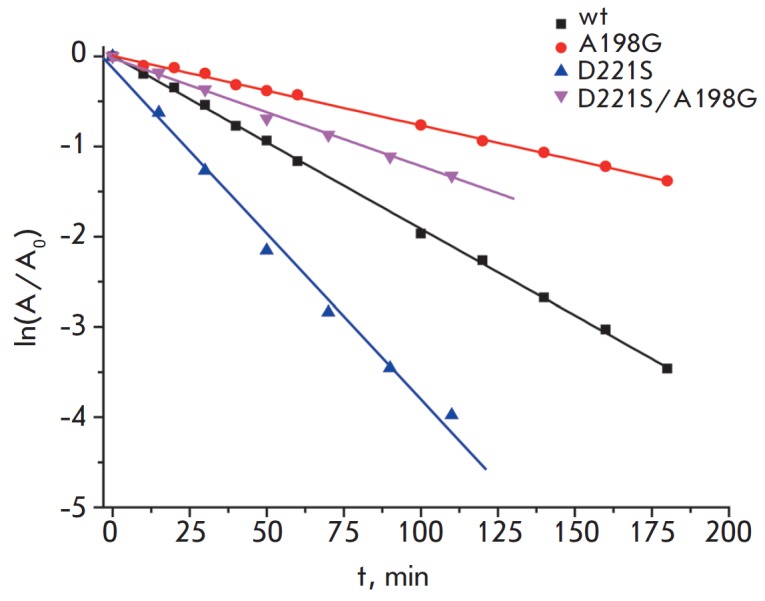
Residual activity as a function of time for mutant PseFDHs and wild-type enzyme, 0.1 M phosphate buffer, pH 7.0, 63oC

The thermostability of mutant PseFDH and MorFDH was determined based on inactivation kinetics at several temperatures. In wild-type PseFDH and its mutants, the temperature range of measurements was 60–65oC (Table 1). The time course of loss of enzymatic activity fits the first-order reaction kinetics for the entire temperature range (*Fig. 4*). The thermal inactivation rate constants were determined as the slope of these lines. The observed inactivation rate constant does not depend on enzyme concentration for the entire temperature range, which means that the inactivation is, in fact, a true monomolecular process. The thermal inactivation rate constants are shown in Table. 1. The stability of mutant PseFDH with Ala198Gly substitution at all temperatures was 2–2.5 times higher than the stability of the wild–type enzyme. A similar effect of increased thermal stability was observed for the pair of native and mutant MorFDHA198G, but due to the fact that MorFDH is 25 times less stable than PseFDH [11] the inactivation kinetics were studied at lower temperatures (56–62oC).

Mutant NADP^+^-specific PseFDH Asp221Ser was less stable than the initial NAD^+^-dependent PseFDH (*Fig. 4* and *Table 1*). Ala198Gly substitution in PseFDH Asp221Ser resulted in a significant improvement in thermal stability; the stabilization effect was even slightly higher than that for a similar substitution in the wild-type (*Table 1*). These data indicate that the methyl group of the Ala198 residue is an important destabilizing factor in this portion of the protein globule. A similar stabilizing effect was observed for Ala198Gly substitution in MorFDH (not shown), which decreased the thermal inactivation rate constant by 2.5 times.

**Table 1:** Inactivation rate constants and activation parameters of mutant PseFDHs and wild-type enzyme

Mutant/T, °C	*k_in_*, 10^-5^*s^-1^				ΔH^≠^, kJ/ mol	ΔS^≠^, J/ (mol*K)
	60.1	62.0	63.0	65.0		
wt-PseFDH	5.4±0.2	22±2	32±2	140±12	570±20	1390±70
PseFDH A198G	2.7±0.1	9.3±0.5	13±0.8	60±5	580±30	1410±80
PseFDH D221S	9.2±0.5	32±4	69±7	188±15	570±40	1410±100
PseFDH D221S/ A198G	2.7±0.1	8.9±0.4	20±3	52±6	580±30	1380±110

**Fig. 5:**
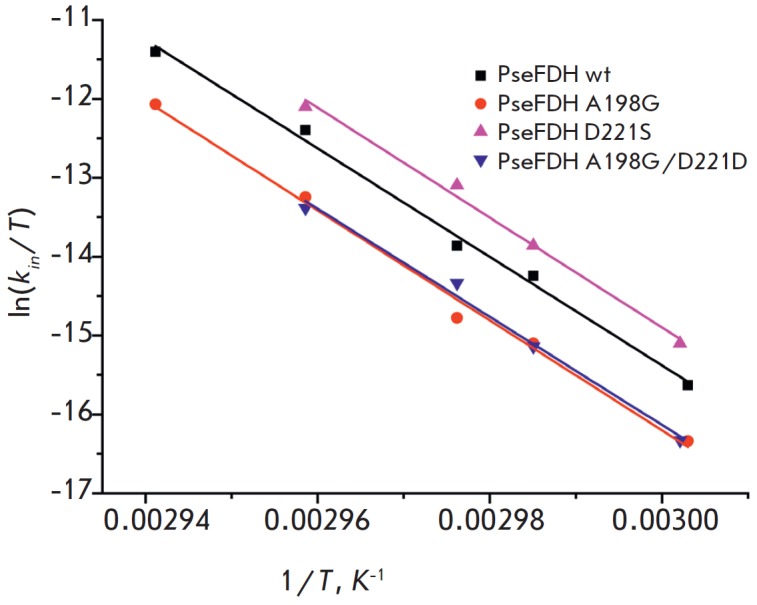
Temperature dependence of the inactivation rate constant in coordinates ln(*k_in_*/*T*) ‑ 1/*T *for mutant PseFDHs and the wild-type enzyme

We were interested in establishing which of the two factors, the change in enthalpy or entropy, increased stability of the obtained mutants. For this purpose, we analyzed the temperature dependence of inactivation rate constants. *Fig. 5* shows the dependence of the first-order inactivation rate constants *k_in_*in coordinates ln(*k_in_*/*T*) vs. 1/*T*, where *T *is the absolute temperature in K.

The linear dependence of the secondary plots suggests that the process of thermal inactivation of native and mutant FDHs is described by the temperature dependence of the rate constant according to the trasition state theory [12]. This equation can be presented in the following linear form:

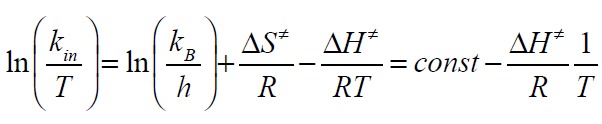



where *kB *and *h *are the Boltzmann and Plank constants, respectively; *R *is the universal gas constant; Δ*H*≠ and Δ*S≠* are activation parameters.

Parallel lines in Fig. 5 indicate that the process of thermal inactivation of wild-type PseFDH and its mutants is characterized by approximately equal *ΔH≠ *values, while the main contribution to increased FDH stability resulting from the introduction of the A198G mutation was determined by the entropy factor. The numerical values of the activation parameters *ΔH^≠^*and *ΔS^≠^*of the thermal inactivation process are shown in Table. 1.

It should be noted that the 2.5-fold increase in enzyme stability resulting from point mutation is a significant effect. Previously, we conducted site-directed mutagenesis experiments with another amino acids residues of the PseFDH globule with non-optimal values of the ψ and φ angles. In these experiments, no increased stability was achieved, since these residues were involved in the formation of hydrogen bonds and the energy of these bonds exceeded the energy of destabilization due to a non-optimal conformation [13].

**Kinetic properties of the mutant enzymes**

The kinetic parameters of the obtained mutants are shown in Table 2. For the sake of comparison, the same table shows similar parameters for other known FDHs. As mentioned above, the results of a computer simulation showed that the introduction of Ala198Gly substitution provides higher mobility to the coenzyme-binding αA-helix domain. As can be seen from Table 2, the introduction of this substitution in NAD^+^-specific wildtype PseFDH and MorFDH improves binding of the coenzyme: in both cases, the Michaelis constant for NAD^+^ decreased by almost twofold, while that for formate remained virtually unchanged. The Michaelis constant is typically not an equilibrium constant; however, the reaction catalyzed by PseFDH and MorFDH has a random quasi-equilibrium kinetic mechanism [11, 19, 20]. In this case, the *K*_M_ value for NAD^+^ and formate is as an equilibrium constant of substrate binding to the corresponding binary complex.

**Table 2:** Kinetic parameters of native and mutant formate dehydrogenases

Enzyme*	K_MK_NAD^+^, μM	K_M_NADP^+^, μM	K_M_^HCOO-^, mM	*k_cat_*, s^-1^	*k_cat_*/K_m_NAD^+^, mM^-1^s^-1^	*k_cat_*/K_m_, mM^-1^s^-1^ mut,/wt,	reference
Reaction with NAD^+^							
wt-PseFDH	60 ± 5		6.5 ± 0.2	7.3 ± 0.2	122	1	[14]
PseFDH A198G	35 ± 2		7.5 ± 0.2	7.3 ± 0.1	209	1.713	Present work
PseFDH D221S	710 ± 45		32 ± 2	5.0 ± 0.3	7.04	0.058	Present work
PseFDH D221S / A198G	540 ± 42		53 ± 1	5.0 ± 0.2	9.26	0.076	Present work
MorFDH	80 ± 7		7.7 ± 0.3	7.3 ± 0.1	91.3	1	[14]
MorFDH A198G	45 ± 3		8.0 ± 0.5	7.3 ± 0.3	162	1.774	Present work
BstFDH	1430		≥ 150	1.7 ± 0.1	1.19	--	[15]
wt-CmeFDH	55		NA	1.4	25.5	--	[16]
CmeFDH D195S	4700		7.0	1.6	0.34	--	[16]
wt-CboFDH	15		5.9	3.7	246.7	--	[17]
CboFDH D195S	1500		NA	0.34	0.227	9.2*10-4	[17]
CboFDH D195N	5010		NA	0.21	0.04	1.7*10^-4^	[17]
CboFDH D195A	4800		NA	0.76	0.158	6.4*10^-4^	[17]
CboFDH D195Q	960		NA	0,26	0.271	0.001	[17]
SceFDH	36		5.5	6.5	181	--	[18]
SceFDH D196A / Y197R	7600		1000	0.095	0.0125	--	[18]
Reaction with NADP^+^							
wt-PseFDH		100000*	NA	1.3 ± 0.1	0.013	1	Present work
PseFDH D221S		190 ± 30	43	1.7 ± 0.2	3.04	234	[7]
PseFDH D221S / A198G		280 ± 25	89	1.8 ± 0.2	6.43	495	Present work
wt-BstFDH		160	55.5	4.75	29.7	--	[15]
CmeFDH D195S		> 0.4 M	NA	NA	NA	--	[16]
wt-CboFDH		> 38000	NA	4*10^-5^	10^-6^	--	[17]
CboFDH D195S		6200	NA	0.34	0.055	55000	[17]
CboFDH D195N		13200	NA	0.26	0.0196	19600	[17]
CboFDH D195A		3300	NA	0.052	0.0157	15700	[17]
CboFDH D195Q		4500	NA	0.26	0.058	58000	[17]
SceFDH D196A/Y197R		4500	1000	0.13	0.03	--	[18]

*PseFDH, MorFDH, BstFDH, CmeFDH, CboFDH, SceFDH – formate dehydrogenases from bacteria Pseudomonas sp.101, Moraxella sp. C-1, Burkholderia stabilis 15516, yeasts Candida methylica and Candida boidinii, and baker’s yeast Saccharomyces cerevisiae, respectively.

Asp221Ser substitution deteriorates the enzyme affinity for NAD^+^ and increases affinity for NADP^+^ (Table 2). This is easily explained by the fact that such a substitution removes the carboxyl group, which provides the specificity to NAD^+^ due to the presence of hydrogen bonds with the 2’- and 3’-OH-groups of the ribose present in adenosine, and repulses the negatively charged 2’-phosphate group of NADP^+^.

Ala198Gly substitution in mutant NADP^+^-specific PseFDH Asp221Ser leads, as in the case of the wildtype enzyme, to improved affinity of the enzyme for NAD^+^ by approximately 1.5 times, but in the case of NADP^+^ affinity for coenzyme decreases by approximately the same value (Table 2). In addition, Ala198Gly substitution in the above-mentioned mutant caused a significant deterioration in the affinity for formate. This was due to the fact that the major structural changes in FDH protein globules required for the formation of the ternary enzyme-substrate complex and the transition state of enzymatic reactions occur at the stage of coenzyme binding. Therefore, the conformation of the double complex (FDH-coenzyme) is critical for efficient binding of formate. It is known that even small changes in the active site of FDH lead to significant changes in the affinity for formate. Data for FDH from bacterium *Burkholderia stabilis *are shown in Table 2. It can be seen that the substitution at position 221 of PseFDH allows one to approach the values of the constants for this natural NADP^+^-dependent enzyme.

Our data allow us to propose a hypothesis explaining the presence of the “non-canonical” Ala residue in the first position of the highly conserved “canonical”sequence GxGxxG for the coenzyme binding domain of PseFDH. Apparently, this enzyme is an intermediate product of formate dehydrogenase evolution from NADP^+^- to a NAD^+^-specific enzyme. During the early stages of development of living systems, when aerobic processes were the main metabolic pathways, FDH was probably an NADP^+^-dependent enzyme and was responsible for the production of NADPH for the biosynthesis of various compounds. The presence of the Ala residue at position 198 in NADP^+^-dependent enzymes provided 10 times more efficient binding of formate than in the case of Gly198. In this enzyme, an uncharged amino acid resided at position 221 and a positively charged Arg residue, required to neutralize the negative charge of the phosphate groups of NADP^+^, was at position 222. An enzyme effectively using NAD^+^ rather than NADP^+^ was required in the course of evolution when methylotrophic microorganisms appeared. The first stage of the evolutionary process was the emergence of the Asp residue at position 221, providing NAD^+^ specificity. FDH from bacterium *Pseudomonas* sp. 101 is probably the product of this evolution stage. This enzyme has an Asp residue responsible for NAD^+^ specificity, while containing a positively charged Arg222 residue involved in the binding of NADP^+^, and Ala at position 198 as “rudiments.” FDH of methylotrophic yeast appeared at the later stages of evolution. These enzymes have lost their “rudimentary” residues and possess absolute specificity for NAD^+^ (*Table 2*). Over the course of evolution, the “non-canonical” Ala residue at position 198 in yeast formate dehydrogenases was replaced by “canonical” Gly and the residue corresponding to Arg222 in PseFDH was substituted by the Tyr residue, as was clearly shown in our experiments studying the changes in coenzyme specificity from NAD^+^ to NADP^+^ in FDH from baker’s yeast [18]. The experiments of changes in FDH coenzyme specificity are described in more detail in [7].
